# Recurrent Rhinosporidiosis: Coblation Assisted Surgical Resection—A Novel Approach in Management

**DOI:** 10.1155/2014/609784

**Published:** 2014-12-10

**Authors:** Iram Khan, Shweta Gogia, Alok Agarwal, Ajay Swaroop

**Affiliations:** Sir Ganga Ram Hospital, New Delhi 110060, India

## Abstract

Recurrent rhinosporidiosis is a chronic granulomatous disease with a known tendency to reoccur. Coblation EVAC 70 is a novel surgical tool which seems to provide excellent option in management of this notorious disease. We present an interesting case and the innovative approach in its management, using Coblation system. *Introduction*. Rhinosporidiosis seeberi causes a chronic granulomatous disease of upper airway, usually involving the nose and nasopharynx, and has a notorious tendency to reoccur. The current line of management is surgical excision of the lesion along with cauterization of the base, which does not prevent reoccurrence of the disease. *Case Presentation*. A 65-year-old male resident of rural India reported a history of breathing difficulty and change in voice. Patient is a Hindu priest by profession, who according to their rituals has to take bath in local pond or river. *Conclusion*. Rhinosporidiosis is a difficult to treat pathology due to its tendency to reoccur. Till date the management of the disease is far from satisfactory. Coblation system which has already found its roots in otorhinolaryngology can be used as a novel tool in surgical resection of recurrent rhinosporidiosis and has added advantage of low temperature dissection along with clear surgical field due to constant suctioning.

## 1. Introduction

Rhinosporidiosis is a rare chronic granulomatous disease of mucocutaneous tissue. This disease is widely endemic in Indian subcontinent and some parts of Africa. The etiological agent rhinosporidiosis seeberi is an aquatic protistan parasite [1]. The disease most commonly involves nasal cavity but can also be seen to involve nasopharynx, larynx, soft palate, skin, bulbar and palpebral conjunctiva, lacrimal sac, nasolacrimal duct, and external urethral meatus [2]. In nasal cavity, it presents as a fleshy bleeding mass which can sometimes be confused with malignancy. The dilemma is in the management of the disease, which has a notorious tendency to reoccur. We report a case of recurrent rhinosporidiosis who presented to our centre with complaints of difficulty in breathing. The patient had undergone multiple surgeries in the past including excision with KTP laser. The patient presented with huge fleshy mass filling both the nasal cavities along with nasopharyngeal and oropharyngeal extension. We surgically excised the mass using Coblation system. Follow-up endoscopic evaluation 1 year after the surgery showed no signs of reoccurrence.

## 2. Case Presentation

A 65-year-old male presented to us with difficulty in breathing, change in voice, and nasal obstruction. Patient was a Hindu priest by profession, was a resident of Jharkhand, and had a positive family history of rhinosporidiosis in a paternal cousin and maternal uncle. Patient had been operated on 11 times earlier; first surgery was done when he was 16 years of age. The last procedure was done about 1 year back with KTP laser when he had to undergo a tracheostomy because of the extensive nature of the disease. On examination, a fleshy mass was seen filling both nasal cavities with yellowish pinhead sized spots on surface. Note was also made of the presence of septal perforation in the cartilaginous part of the septum. The mass was extending in the oropharynx behind the soft palate. On laryngoscopy, growth was seen involving bilateral vallecula, bilateral glossoepiglottic fold, and lingual surface of epiglottis pushing the epiglottis over the laryngeal inlet. Vocal cords could not be visualized. A contrast enhanced computed tomographic scan was done, which revealed a homogenous mass filling bilateral nasal cavity without any bony invasion, although thinning of bilateral lamina papyracea was noted. Also mass was filling nasopharynx and oropharynx involving the lingual surface of epiglottis, obscuring laryngeal inlet (Figure 2). Taking into account his history and the severity of his symptoms, a decision was made to operate on the patient.

**Figure 1:**
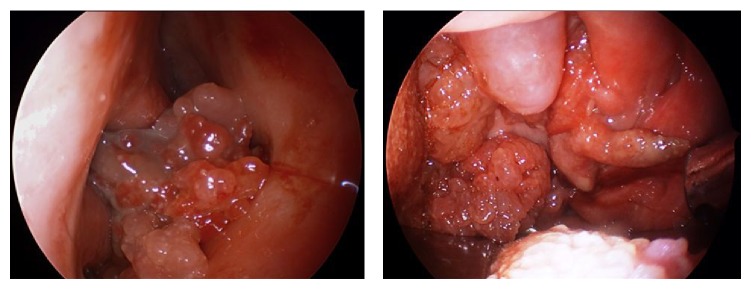
Fleshy papillary mass filling nasal cavity and oropharynx.

**Figure 2:**
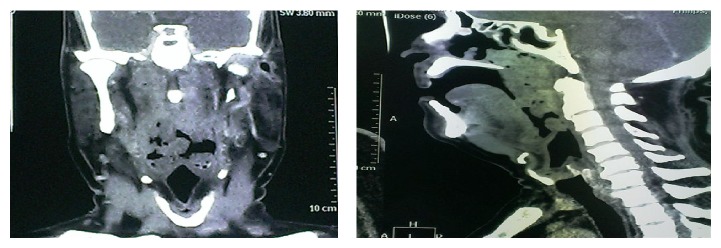
CT scan coronal and sagittal section views showing extension of the tumor in nasopharynx and oropharynx.

An elective tracheostomy was done taking into view the difficult airway. Patient was put under general anesthesia. The nasal extension of the mass was first addressed under endoscopic vision. Using Coblation wand EVAC 70, the nasal mass was ablated but there was no attachment in the nasal cavity and mass appeared to arise from the nasopharynx attached to the posterior wall. The patient was then put in tonsillectomy position and mouth gag was applied and then mass was removed from the nasopharynx using Coblation wand at a setting of 8 in ablation mode, and the wand had to be bent to approach the choanal region. Following this the mass was removed from the oropharynx including vallecula and lingual surface of epiglottis and lateral glossoepiglottic folds (Figure 1). To stop bleeding coagulation mode was used intermittently at a setting of 4. Two wands of EVAC 70 were used and the total operating time was 50 mins. The patient was decannulated on third postoperative day and was discharged on fifth postoperative day.

On gross pathology, fleshy mass with white pinhead sized spots on the surface was noted. Microscopic evaluation identified sporangia and endospores demonstrating a granulomatous reaction with eosinophilia. Final diagnosis was consistent with rhinosporidiosis with chronic inflammation and giant cell reaction. Patient was started also on dapsone after assessing the G6PD level on 100 mg twice daily.

Postoperatively patient was followed up for 1 year, during which patient was subjected to endoscopic examination twice, with no evidence of reoccurrence at 1 year of follow-up.

## 3. Discussion

Rhinosporidiosis is a rare disease which has been known for hundred years since its first description in 1900 by Seeber, an individual from Argentina [2]. It is a chronic disease with frequent reoccurrence, usually involving upper respiratory sites. The disease is known to be endemic in South Asia, primarily in southern India and in Sri Lanka [2]. The high reason of endemicity could be because of people bathing in stagnant waters; in addition, the other aquatic microorganisms might be a synergistic factor. Most common site involved is traumatized epithelium which is rampantly seen in nose [2]. This could explain occurrence of disease in river-sand workers. Another mode of infection is autoinoculation which was first described by Karunarate which explains occurrence of satellite lesion adjacent to granulomas [3].

Rhinosporidiosis seeberi, the causative organism, has a debatable taxonomy as the microorganism is intractable to isolation and microbiological culture, and the morphological features resemble both fungi and protozoa. But recently a study has classified* Rhinosporidium seeberi* in a class, the Mesomycetozoea, along with 10 parasitic and saprobic microbes. The controversial spherical bodies have been shown to comprise both lipid-protein nutritive bodies and other spherical bodies [4]. This is supported by a study conducted by Herr et al. group which includes fish and amphibian pathogens in the form of DRIP clade (*Dermocystidium*, the rosette agent,* Ichthyophonus*, and* Psorospermum*) [4, 5].

Diagnosis is made by biopsy and histological examination of the section. The tumor is covered with columnar epithelium, along with many spores and sporangia in various stages of development. Also there is cellular infiltrate with lymphocytes, plasma cells, polymorphs, and eosinophils [6].

The treatment of choice is wide local excision with cauterization of the base. In addition, systemic dapsone is given for a period of 1 year and is known to prevent reoccurrence which arrests the maturation of sporangia and promotes fibrosis [2, 7, 8]. But very few studies were found in literature reporting the management of recurrent rhinosporidiosis.

In a case report of recurrent rhinosporidiosis by Nichlani, in which they excised the lesion using diode laser, they followed up the case for 1 year, in which no recurrence was reported [9]. In 2014 a case has been reported by J. Chery, C. Bacskai, and E. Mendoza in which they have excised the recurrent rhinosporidiosis with harmonic scalpel, citing the superior hemostasis of the instrument while preserving the local integrity of local tissue [10]. In a prospective study by surgical excision with electrocautery, can cause localized tissue trauma and in addition expose the unaffected sites to the blood during surgery, which in turn can cause inoculation. This possibly explains the recurrence in our case.

Coblation has recently been used to treat various otolaryngological conditions: obstructive sleep apnea, tonsillectomy, turbinate reduction, and benign tumors of head and neck [8]. Coblation, a radiofrequency energy, has been reported to penetrate surrounding tissues to a depth of only 100 *μ*m [11]. As the temperature does not exceed 60°C in Coblation mode, there is less thermal damage to surrounding tissues as seen with cautery and laser. Also, there is constant cooling due to simultaneous irrigation.

**Figure 3:**
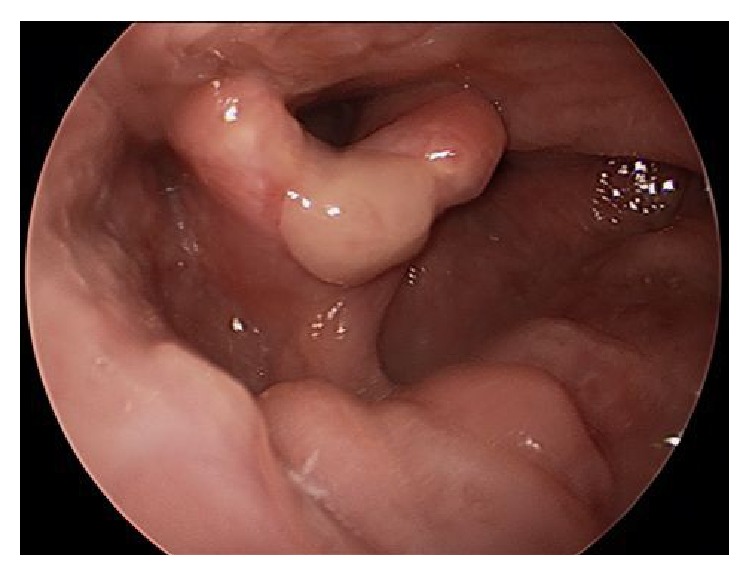
Post-op follow-up picture after 1 year shows no signs of reoccurrence.

With the use of Coblation system, we could achieve complete resection of the lesion, with minimum bleeding and less blood contamination, also preserving the integrity of local tissue. Patient is under follow-up for last 1 year and there is no evidence of occurrence (Figure 3).

## 4. Conclusion

Coblation system can be a promising new tool in the surgical resection of recurrent rhinosporidiosis. This system enables the surgeon to obtain better resection of the disease due to constant suctioning, with minimum lateral thermal damage. This also decreases the chances of reoccurrence due to very little contamination of the surrounding areas by preventing autoinoculation. The novel design of the wand can help to resect “the difficult to reach areas.”
